# Microarray analysis of kiwifruit (*Actinidia chinensis*) bark following challenge by the sucking insect *Hemiberlesia lataniae* (Hemiptera: Diaspididae)

**DOI:** 10.1016/j.gdata.2016.01.016

**Published:** 2016-02-02

**Authors:** M. Garry Hill, Kirstin V. Wurms, Marcus W. Davy, Elaine Gould, Andrew Allan, Nicola A. Mauchline, Zhiwei Luo, Annette Ah Chee, Kate Stannard, Roy D. Storey, Erik H. Rikkerink

**Affiliations:** a New Zealand Institute for Plant & Food Research Limited (PFR), Canterbury Agriculture & Science Centre, Gerald St, Lincoln 7608, New Zealand; b PFR, Private Bag 3230, Waikato Mail Centre, Hamilton 3240, New Zealand; c PFR, 412 No1 Rd RD2, Te Puke, New Zealand; d Zespri International Limited, 400 Maunganui Road, Mount Maunganui 3116, New Zealand; e PFR, Private Bag 92169, Auckland, New Zealand

## Abstract

Both commercial and experimental genotypes of kiwifruit (*Actinidia* spp.) exhibit large differences in response to insect pests. An understanding of the vine's physiological response to insect feeding and its genetic basis will be important in assisting the development of varieties with acceptable levels of pest resistance. This experiment describes transcriptome changes observed in the bark of kiwifruit 2 and 7 days after the commencement of feeding by the armored scale insect pest, *Hemiberlesia lataniae*. Using a cDNA microarray consisting of 17,512 unigenes, we measured transcriptome changes and analyzed these into functional ontology categories using MapMan. Results are available in the GEO database GSE73922 and are described fully in Ref. Hill et al. (2015) [1]. After 7 days, transcripts associated with photosynthesis were down-regulated and secondary metabolism was up-regulated. Differential expression of transcripts associated with stress response was consistent with a defense response involving both effector and herbivore-triggered immunities, with predominant involvement of the salicylic acid phytohormonal pathway. This hypothesis was supported by the results of two laboratory experiments. The methods described here could be further adapted and applied to the study of plant responses to a wide range of sessile sucking pests.

**Table:** 

Specifications	
Organism/cell line/tissue	*Actinidia chinensis* Planch var. *chinensis* ‘Hort16A’
Sex	Female
Sequencer or array type	*Actinidia chinensis* spotted oligonucleotide microarray
Data format	Microarray raw and normalized data as .gpr files
Experimental factors	Kiwifruit canes with and without application of *Hemiberlesia lataniae* neonate ‘crawlers’, sampled 2 days and 7 days after application of the insects. Six biological replicates each duplicated twice in the microarray.
Experimental features	Crawlers (neonate nymphs) from laboratory cultures applied to 9-week-old canes. Bark was removed after 2 and 7 days and RNA extracted.
Consent	N/A
Sample source location	Plant & Food Research Institute, Research Orchard, Te Puke, New Zealand

## 1 Direct link to deposited data

http://www.ncbi.nlm.nih.gov/geo/query/acc.cgi?acc=GSE73922.

## 2 Experimental design, materials and methods

### 2.1 Plant material

Eighteen 2-year-old clonal *Actinidia chinensis* ‘Hort16A’ scions were grafted onto 2-year-old clonal *A. polygama* ‘Kaimai’ rootstocks in December 2005 at the Plant & Food Research, Te Puke Research Orchard, Te Puke, New Zealand. Vines were uprooted in July 2007, potted in 30-liter planter bags, pruned and held in a shade house (50% shade) under ambient conditions for 15 months prior to the experiment. New cane development (bud break) began in the third week of August 2008. The canes were 9 weeks old (last week of October 2008) at the commencement of the experiment.

### 2.2 Insect material

Female *Hemiberlesia lataniae* (only unisexual populations exist in New Zealand) were reared as pure cultures on washed, commercially sourced potato and squash in a constant environment room (20–21 °C, 60–80% R.H. and ambient day length). The insects were sourced from a colony derived from insects collected in approximately 2002 from kiwifruit vines growing on the Plant & Food Research, Te Puke Research Orchard. The insects mature after 9–10 weeks and are reproductive for a further 6–10 weeks, each producing ~ 50–100 offspring. First instar crawlers emerge from beneath the female scale cap in the first 4–6 h of the photophase. The crawler stage lasts for less than 1 day before the insect settles to feed, spins a waxy cap over itself and becomes sessile [2]. For further information on armored scale rearing and biology see Ref. [3].

### 2.3 Experimental conditions and methods

In the last week of October 2008, 9 weeks after bud break, two actively growing canes (minimum 40 cm long) were chosen on each vine. A length of approximately 20 cm was selected on each cane and wool yarn was wrapped loosely around the cane at a spacing of approximately 1 cm to assist crawler settlement. The selected area on both canes of 12 of the vines was seeded with 150 *H. lataniae* crawlers (< 1-day old) between 1.00 and 3.00 pm on 10 November 2008, using a soft paintbrush to transfer crawlers individually from the insect colony onto each cane. The remaining 12 vines, with wool-wrapped canes but no insects, were used as controls.

Six of the twelve *H. lataniae* treatment vines and six of the control vines were chosen at random and the bark within the area occupied by the settled insects was sampled after 2 days (12 November 2008 at 1.00–3.00 pm) and 7 days (17 November 2008 at 1.00–3.00 pm). The wool yarn crawler settlement aids were removed and the number of settled first instar insects (white caps) was quickly counted. The wool was left until the bark was sampled because its removal can cause damage to the delicate scale caps covering the insects. A 7 cm length of bark was removed from each cane (two per vine) within the region settled by the insects, by making an incision along the length of the cane down to the cambium layer using a scalpel and peeling the bark from the cane with fingers. The bark with its associated scale insects was placed immediately into a labeled plastic vial in liquid nitrogen. Bark removal was completed within 30 s from bark incision to immersion in nitrogen, for each sample. The samples were stored at − 80 °C until extraction approximately 2 months later. Atmospheric conditions in the shade house, measured by data loggers, varied from 21 to 31 °C and 40–75% RH during the experiment.

### 2.4 RNA extraction

Total RNA (mean 26 ± 2.5 (SE) μg/ml) was extracted from 1.5 to 2.5 g of frozen kiwifruit bark using the method of Chang et al. [4] which is suitable for samples containing high levels of polyphenols and polysaccharides. Initially plant tissue was ground to fine powder by hand using liquid nitrogen in a mortar and pestle, followed by placement of the cane material into warmed (65 °C) extraction buffer [4] and a further 1–2 min of grinding using a polytron (PT3000, Kinematica AG, Lucerne, Switzerland) to ensure complete homogenization and release of cellular contents.

RNA samples were quantified, and sample purity was verified by using a Nanodrop ND-1000 spectrophotometer (Thermo Fisher Scientific), where 260:280 absorbance ratios of 1.8–2.0 were considered of acceptable purity. RNA integrity was checked by an Agilent 2100 analyzer (Agilent Technologies), where non-degraded RNA gave sharp 18S and 28S bands.

### 2.5 Gene expression and data analysis

The microarray chips consisted of 17,512 oligonucleotide probes, 45–55 bases long, with a consistent melting temperature. The chip was designed using sequence data from an *Actinidia* expressed sequence tag (EST) library [5]. At the time of synthesis of the microarray chip, the EST database comprised 41,858 non-redundant clusters consisting of 132,577 ESTs. The chip was designed for the analysis of fruit characteristics and not plant resistance. cDNA synthesis, labeling, and hybridization were carried out according to Janssen et al. [6]. Microarray images were converted to 16-bit intensities using GenePix 4000B scanner and Genepix Pro 4.0 software (Molecular Devices). Genepix intensity files were preprocessed and analyzed using the limma [7] analysis package in Bioconductor [8]. Data were normalized using the print tip LOESS method [9], and candidate differentially expressed genes (DEGs) identified using a false discovery rate control set at P < 0.05 [10].

Validation of microarray analysis was carried out by measuring the gene expression of six genes using real time qPCR and comparing the results with the microarray data. The genes were selected to provide a range of up- and down-regulated values in the microarray. The microarray expression values were compared with those obtained with the qPCR. Total RNA extracted from kiwifruit bark was treated with deoxyribonuclease I (DNase) (Amplification Grade kit; Invitrogen Catalog No. 18068-015), to remove any genomic DNA and checked by PCR to confirm that there was no genomic contamination. First-strand cDNA was synthesized in a 20 μl reaction volume containing 2 μg of DNase-treated RNA, using the Super-Script III first-strand synthesis system for RT-PCR (Invitrogen Catalog No. 18085-051). Non-template controls included in each PCR plate were used to check the purity of the reagents. qPCR was performed in triplicate on RNA from three biological replicates (vines) in 10 μl reactions containing 1 μl of the cDNA (diluted 10-fold in water), 1 μM of each of forward and reverse primers and 5 μl of Light Cycler® 480 SYBR Green 1 Master Mix (Roche Diagnostics GmbH, Mannheim, Germany, Product No. 04 887 352 001). The primers were designed using Primer3 software (Whitehead Institute, Cambridge, MA, USA) and were synthesized by Invitrogen (Auckland, New Zealand). qPCRs were carried out in a Corbett Rotor-Gene™ 6000 system (Corbett Life Science, Concorde, NSW, Australia). The relative quantification thermal cycling conditions were: denaturation at 95 °C for 10 min; followed by 45 cycles of 10 s denaturation at 95 °C, 5 s annealing at 55 °C and 20 s extension at 72 °C. Melting curve analysis (60–95 °C at 1 °C increments with 5 s between each step) was performed after the final qPCR cycle to validate amplicon specificity. Two reference genes that were stably expressed under the conditions of the experiment, actin and elongation factor (EF) were used for normalization. A gene expression normalization factor was calculated for the relative expression of each target gene using geNorm v3.5, based on the geometric mean of actin and EF. Expression of the *H. lataniae* treatment is expressed relative to the challenged control RNA samples, which were assigned a value of 1.

MapMan software [11], [12] was used to categorize transcripts into functional categories, and additional manual categorization was carried out to reveal more stress-related transcripts. Transcripts associated with photosynthesis were down-regulated while those associated with secondary metabolism were up-regulated. One hundred stress-related transcripts were identified [13], providing useful initial insights into the plant's response to *H. lataniae* attack.

## 3 Discussion

The results of this microarray experiment have identified that, in response to attack by *H. lataniae*, the bark of the kiwifruit plant down-regulated genes involved in photosynthesis and up-regulated genes involved in secondary metabolism, including the production of a range of defense-related compounds, in particular those associated with the phenylpropanoid pathway. There was evidence for both effector-triggered and hamp-triggered (herbivore-associated molecular pattern-triggered) immune responses using the salicylic acid pathway as a major phytohormonal defense response. As a preliminary experiment investigating the transcriptomic response of kiwifruit bark to armored scale insect attack, this work has successfully demonstrated a broad range of responses related to stress, photosynthesis and secondary metabolism. On the basis of this study, we can design further experiments focusing on particular stress- and defense-related genomic responses of interest.

When considering a cut-off for significant transcriptome effects, we chose a modest fold-change of 1.5 (log_2_(0.585); where P < 0.05) on the basis that the quantity of plant tissue challenged by each tiny neonate insect (~ 0.4 mm long) was very small and the density of insects was quite low (estimated 0.3–1.0 cm^2^ of bark tissue per insect), and thus the overall quantity of the affected tissue in each sample was likely to be low. Kiwifruit has a large and still relatively poorly understood genome [14], and the only microarray chip available was designed to investigate fruit characteristics such as flavor, fragrance, convenience, appearance and nutritional qualities [5]. In spite of these limitations, the experiment was able to identify a large number of defense- and stress-related gene orthologs, showing that this experimental technique and somewhat dated microarray technology may be useful for providing initial insights into transcription responses involving sessile sucking pests that have hitherto received little study.
